# Long-read genome assembly of the Japanese parasitic wasp *Copidosoma floridanum* (Hymenoptera: Encyrtidae)

**DOI:** 10.1093/g3journal/jkae127

**Published:** 2024-06-11

**Authors:** Kouhei Toga, Takuma Sakamoto, Miyuki Kanda, Keita Tamura, Keisuke Okuhara, Hiroko Tabunoki, Hidemasa Bono

**Affiliations:** Laboratory of BioDX, PtBio Co-Creation Research Center, Genome Editing Innovation Center, Hiroshima University, 3-10-23 Kagamiyama, Higashi-Hiroshima city, Hiroshima 739-0046, Japan; Laboratory of Genome Informatics, Graduate School of Integrated Sciences for Life, Hiroshima University, 3-10-23 Kagamiyama, Higashi-Hiroshima city, Hiroshima 739-0046, Japan; Department of Science of Biological Production, Graduate School of Agriculture, Tokyo University of Agriculture and Technology, 3-5-8 Saiwai-cho, Fuchu, Tokyo 183-8509, Japan; Laboratory of BioDX, PtBio Co-Creation Research Center, Genome Editing Innovation Center, Hiroshima University, 3-10-23 Kagamiyama, Higashi-Hiroshima city, Hiroshima 739-0046, Japan; Research and Development Department, PtBio Inc., 3-10-23 Kagamiyama, Higashi-Hiroshima city, Hiroshima 739-0046, Japan; Laboratory of BioDX, PtBio Co-Creation Research Center, Genome Editing Innovation Center, Hiroshima University, 3-10-23 Kagamiyama, Higashi-Hiroshima city, Hiroshima 739-0046, Japan; Laboratory of Genome Informatics, Graduate School of Integrated Sciences for Life, Hiroshima University, 3-10-23 Kagamiyama, Higashi-Hiroshima city, Hiroshima 739-0046, Japan; Research and Development Department, PtBio Inc., 3-10-23 Kagamiyama, Higashi-Hiroshima city, Hiroshima 739-0046, Japan; Department of Science of Biological Production, Graduate School of Agriculture, Tokyo University of Agriculture and Technology, 3-5-8 Saiwai-cho, Fuchu, Tokyo 183-8509, Japan; Cooperative Major in Advanced Health Science, Graduate School of Bio-Applications and System Engineering, Tokyo University of Agriculture and Technology, 3-5-8 Saiwai-cho, Fuchu, Tokyo 183-8509, Japan; Laboratory of BioDX, PtBio Co-Creation Research Center, Genome Editing Innovation Center, Hiroshima University, 3-10-23 Kagamiyama, Higashi-Hiroshima city, Hiroshima 739-0046, Japan; Laboratory of Genome Informatics, Graduate School of Integrated Sciences for Life, Hiroshima University, 3-10-23 Kagamiyama, Higashi-Hiroshima city, Hiroshima 739-0046, Japan

**Keywords:** *Copidosoma floridanum*, Pacific Biosciences high-fidelity reads, genome assembly

## Abstract

*Copidosoma floridanum* is a cosmopolitan species and an egg-larval parasitoid of the Plusiine moth. *C. floridanum* has a unique development mode called polyembryony, in which over two thousand genetically identical embryos are produced from a single egg. Some embryos develop into sterile soldier larvae precociously, and their emergence period and aggressive behavior differ between the US and Japanese *C. floridanum* strains. Genome sequencing expects to contribute to our understanding of the molecular bases underlying the progression of polyembryony. However, only the genome sequence of the US strain generated by the short-read assembly has been reported. In the present study, we determined the genome sequence of the Japanese strain using Pacific Biosciences high-fidelity reads and generating a highly contiguous assembly (552.7 Mb, N50: 17.9 Mb). Gene prediction and annotation identified 13,886 transcripts derived from 10,786 gene models. We searched the genomic differences between US and Japanese strains. Among gene models predicted in this study, 100 gene loci in the Japanese strain had extremely different gene structures from those in the US strain. This was accomplished through functional annotation (GGSEARCH) and long-read sequencing. Genomic differences between strains were also reflected in amino acid sequences of *vasa* that play a central role in caste determination in this species. The genome assemblies constructed in this study will facilitate the genomic comparisons between Japanese and US strains, leading to our understanding of detailed genomic regions responsible for the ecological and physiological characteristics of *C. floridanum.*

## Introduction

*Copidosoma floridanum* (Hymenoptera: Encyrtidae) is a worldwide distributed egg-larval parasitoid of the Plusiinae moth (Lepidoptera, Noctuidae, Plusiinae) (Guerrieri and Noyes 2005). This species reproduces by polyembryony, producing >2,000 genetically identical individuals from a single egg (Ode and Strand 1995). Polyembryony has evolved independently in the four Hymenoptera families (Encyrtidae, Platygastridae, Braconidae, and Dryinidae); however, encyrtid wasps have the most extreme form regarding clone size (Segoli et al. 2010). Embryos develop into larvae that feed on the host tissues until pupation. In some encyrtid wasps, the embryos develop into two types of larvae: reproductive larvae and sterile soldier larvae. Sterile soldier larvae possess large mandibles and contribute to the defense of their clonal siblings by attacking against a competitor in the host (Cruz 1981; Strand 2003; Iwabuchi 2019). The phenomenon of solitary individuals forming cooperative groups with a reproductive division of labor can be considered as one of the major evolutionary events that brought complexity to life on Earth (Szathmáry and Smith 1995; Whyte 2021).

The process and molecular basis of soldier development in *C. floridanum* have been described and investigated mainly in the US and Japanese strains (Ode and Strand 1995; Smith et al. 2017; Sakamoto et al. 2020). Like other hymenopteran insects (Cook 1993), *C. floridanum* is haplodiploid species in which the females develop from fertilized eggs and the males develop from the unfertilized egg (Iwabuchi 2019). Soldier larvae develop from both male and female embryos, and the development and behavioral patterns of male soldiers differed between intraspecific strains. In the US strain, male soldiers appear in the penultimate larval stage of the host (Grbic et al. 1997), whereas in the Japanese strain, they appear in the first- or second-instar larvae of the host (Yamamoto et al. 2007). Male soldiers were functional in the Japanese strain but were not as aggressive as the female soldiers (Uka et al. 2013). Male soldiers in the US strain are nonaggressive (Giron et al. 2007). *vasa*, encoding a DEAD-box RNA helicase protein (Hay et al. 1988; Lasko and Ashburner 1988), has been shown to play a central role in caste determination of *C. floridanum* (Donnell et al. 2004; Zhurov et al. 2004). The *vasa* gene was originally identified in *Drosophila melanogaster* as a maternal-effect factor for germ-cell specification and development of abdominal segments (Schupbach and Wieschaus 1986), and vasa's orthologs have been found in invertebrates and vertebrates (Raz 2000). *vasa* has been compared between the Japanese and the US strains of *C. floridanum*, and a 10 amino acid sequence difference in the N-terminal region was observed (Ohno et al. 2019).

Each strain should optimize its behavior and developmental patterns for its habitat. Genome or transcriptome analyes should accelerate our understanding of the molecular basis underlying the intraspecific differences in parasitic ability including polyembryony and caste system (Sakamoto et al. 2020). The genome of the US strain has been sequenced using short-read sequencing (i5K Consortium 2013), while that of the Japanese strain has not. Long-read sequencing offers several advantages over short-read sequencing (Pollard et al. 2018). High-quality (high contiguity of the assembly and completeness of gene sets) insect genomes have recently been achieved using long-read sequencing (Hotaling et al. 2021). Single-molecule real-time (SMRT) sequencing developed by Pacific Biosciences (PacBio) employs the DNA polymerase reaction from a single circular DNA template (Eid et al. 2009), generating highly accurate long high-fidelity reads (HiFi reads, > 10 kb, > 99%) (Rhoads and Au 2015; Ardui et al. 2018; Wenger et al. 2019). HiFi reads are more suitable than other long- or short-read methods for identifying genes containing repetitive sequences (Kawahara et al. 2022; Hotaling et al. 2023).

Comparisons among intraspecific genomes should be useful in understanding the various parasitic ability in *C. floridanum*. In this study, we aimed to construct a highly contiguous genome assembly of the Japanese *C. floridanum* using PacBio long-read sequencing. We performed the quality check, the gene prediction, and functional annotation for the de novo assembly. Gene annotation and amino acid sequences of *vasa* were compared to clarify the genomic differences between Japanese and U.S. strains.

## Materials and methods

### DNA extraction and genome sequencing

The host insect, *Thysanoplusia intermixta* larvae, was collected from Kiyose-city and Fuchu-city in Tokyo. *T. intermixta* larvae were maintained at 25°C in a 16-h light/8-h dark photoperiod, as previously described (Sakamoto et al 2020). We then divided the host *T. intermixta* into two categories, parasitized or unparasitized by *C. floridanum* before *C. floridanum* adults emerged. Emergent *C. floridanum* adults were collected from the parasitized *T. intermixta* larva, and parasitism by *C. floridanum* males was achieved by exposing host eggs (aged 8–14 h old) to non-mated parasitoid females. In this study, the adult *C. floridanum* male was from the third generation of cumulative rearing. Approximately 400 adult *C. floridanum* males were collected and used for DNA extraction. Genomic DNA was extracted from adult *C. floridanum* males using the Blood & Cell Culture DNA Maxi Kit (Qiagen Co. Ltd., Valencia, CA, USA). The genetic system of *C. floridanum* and other hymenopteran insects is haplodiploid; females develop from fertilized eggs (diploid), and males develop from unfertilized eggs (haploid). In this study, the male adults were used. The male adults are clones born from a single egg and are genetically identical. The sequence library was prepared using the SMRTbell Express Template Preparation Kit 2.0 or SMRTbell Prep Kit 3.0 (Pacific Biosciences, Menlo Park, CA, USA). Libraries were sequenced using the Sequel II or IIe (Pacific Biosciences).

### *De novo* assembly and quality assessment

Subreads (DNA derived from males collected in Kiyose city) and HiFi reads (DNA derived from males collected in Fuchu city) generated from the PacBio sequencers were assembled using Flye v 2.9 (Kolmogorov et al. 2019) and Hifiasm v 0.16.1 using the “-primary” option (Cheng et al. 2021), respectively. In the PacBio long-read system, polymerase reads are yielded by sequencing circularized DNA. Then, the adaptors are removed from the polymerase reads, offering the subreads (refer to https://www.pacb.com/technology/hifi-sequencing/). HiFi reads are defined as the consensus sequence called from the subreads. Hifiasm v 0.16.1 can produce the primary contig and alternate contig. The primary contig indicates the haplotype and the alternate contig is composed of short haplotype-specific contigs (haplotigs). Quast v 5.2.0 was used to assess the genome assembly statistics (Gurevich et al. 2013). Gene set completeness was assessed using the Benchmark of Universal Single-Copy Orthologs (BUSCOs) v 5.2.2, with “-l insecta_odb10 (2020-09-10)” or with “-l hymenoptera_odb10” (2024-01-08) (Manni et al. 2021). *k*-mer-based validation was performed using Merqury v1.3 and Meryl db (*k* = 21) (Rhie et al. 2020) for the assembly constructed with HiFi reads. Contigs were assigned to taxonomic classifications using BlobTools v 1.1 (Laetsch and Blaxter 2017) with the BLAST (BLASTN) program in the National Center for Biotechnology Information BLAST software package (v 2.14.0) against the NCBI non-redundant nucleotide sequence database (nt), prepared using ncbi-blast-dbs v 10.5.0 (https://github.com/yeban/ncbi-blast-dbs, accessed on May 18, 2023). minimap2 v 2.26-r1175 (Li 2018) was used to obtain mapping coverage.

Dot plots were visualized using the dotPlotly program with “-s -t -m 10000 -q 25000 -l” (https://github.com/tpoorten/dotPlotly, accessed on July 17, 2023) to compare the genomic differences between the US and Japanese strains. The 1 Mb or longer contigs were extracted using SeqKit v 2.0.0, aligned by a number with default settings, and delta files were filtered using a delta-filter with “-q -r” options in MUMmer4 v4.0.0rc (Marçais et al. 2018).

### Gene prediction and functional annotation

Gene prediction was performed using the BRAKER2 and BRAKER3 databases (Brůna et al. 2021; Gabriel et al. 2024). Public RNA sequencing (RNA-Seq) data of morula (Sakamoto et al. 2020: DRR138914, DRR138915, DRR138916, and DRR138917) and male adult: (i5K Consortium 2013: SRR1864697). RNA-Seq labeled by DRR is derived from Japanese strain, and RNA-Seq labeled by SRR is derived from the US strain. OrthoDB protein sets of Arthropoda (Kuznetsov et al. 2023) (https://bioinf.uni-greifswald.de/bioinf/partitioned_odb11/, accessed on June 17, 2023) were used as extrinsic evidence data to perform the BRAKER pipeline. The RNA-Seq reads were processed using fastp v 0.23.2 with default settings (Chen 2023) and mapped to the de novo assembled genome using HISAT2 v 2.2.1(Kim et al. 2019). The generated SAM files were converted to BAM files using SAMtools v 1.12 (Danecek et al. 2021) and the BAM files were imported together when the BRAKER pipeline was run. Repetitive assembly sequences were soft-masked using RepeatModeler v 2.0.3 and RepeatMasker v 4.0.6 in Extensive de novo TE Annotator v 2.1.3 (Ou et al. 2019). The soft-masked genome assembly was inputted into the BRAKER pipeline. GFFRead v0.12.1 (Pertea and Pertea 2020) was used to convert the transcript or protein sequences from the GTF file created by BRAKER. Protein sequences were assessed using BUSCO (protein mode) with “-l insecta_odb10” (2020-09-10).

The functional annotation workflow of Fanflow4Insects (Bono et al. 2022) was modified and used for protein sequences, as described below. The protein sequences were aligned against reference datasets of *Homo sapiens*, *Mus musculus*, *Caenorhabditis elegans*, *Drosophila melanogaster*, *C. floridanum* (US strain), and UniProtKB/Swiss-Prot using GGSEARCH v 36.3.8 g in the FASTA package (https://fasta.bioch.virginia.edu/, accessed on July 19, 2023). Protein sequences were also searched using HMMSCAN in HMMER v 3.3.2. Pfam v 35.0 was used as the protein domain database for HMMSCAN (Mistry et al. 2021). Enrichment analysis was performed using Metascape software (https://metascape.org/, accessed on July 19, 2023) with default settings (Zhou et al. 2019). Structures of exon and intron were visualized using Jbrowse2 (Diesh et al. 2023). Multiple sequence alignments were perfumed by MAFFT (Katoh et al. 2018). We performed TBLASTN with default setting, and performed BLASTP with “-evalue 0.01”.

## Results and discussion

### *De novo* genome assembly of Japanese *C. floridanum* and the quality assessment

**Fig. 1.**
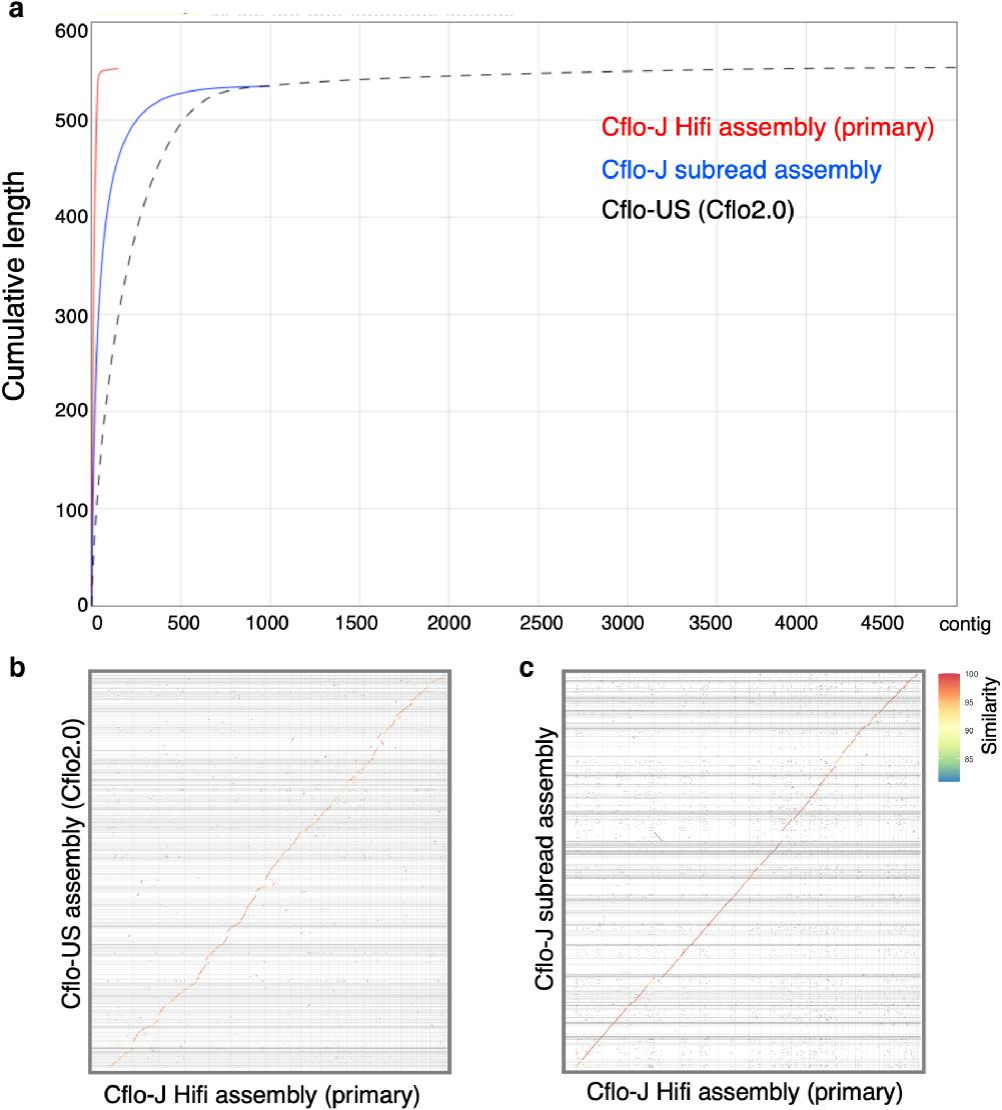
Contiguity and sequence similarity of each assembly. a) Each color shows the cumulative base pair length and number of contigs for each assembly. The assemblies generated from HiFi reads contain the fewest contigs. (b, c) Dot-plot between the Japanese *Copidosoma* strain (Cflo-J) and US *C. floridanum* (Cflo-US) high-fidelity (HiFi) assemblies and between Cflo-J HiFi and Cflo-J subread assemblies. The vertical axis indicates Cflo-US or Cflo-J subread assemblies. The horizontal axis indicates Cflo-J HiFi assembly. The average identity percentages (per query) are displayed in different colors.

This study generated HiFi reads and subreads using Japanese strains (Cflo-J). HiFi reads were assembled, and the primary contig comprised 149 contigs with an N50 length of 17.9 Mb and a total assembly size of 552,713,363 bp (50.8-fold coverage; Table 1 and Supplementary Table 1). Hifiasm also produced alternate contigs (1,162 contigs, Supplementary Table 1). The genome assembly generated by subreads comprised 996 contigs with an N50 length of 4.6 Mb and a total assembly size of 534,638,157 bp (244.7-fold coverage, Table 1 and Supplementary Table 1). Cflo_2.0 (NCBI RefSeq assembly GCF_000648655.2) was used as the genome assembly of US strain (Cflo-US). The assembly quality between the Japanese strain (Cflo-J) and the US strain (Cflo-US) was evaluated. The N50 value of Cflo-J assemblies was much higher than that of Cflo-US (1.2 M). However, the GC content of the assemblies was similar. The BUSCO completeness of Cflo-J was also comparable to that of Cflo-US; however, the fragmentation score was lower for the Cflo-J assemblies (Table 1). The N50 values and number of contigs were the best for the HiFi assembly (Table 1 and Fig. 1a). We confirmed the differences in genomic structures among all the assemblies using dot-plot visualization (Fig. 1b and c). Linear plots were obtained, confirming the similarity of the genomic structures across the entire assembly length (Fig. 1b and c). The matching coordinate data for the dot-plot comparison are available in Supplementary Files 1 and 2.

**Table 1.** Comparison of assembly statistics with the current reference genomes.

	Parameter	Cflo-J Hifi (primary)	Cflo-J subread	Cflo-US (Cflo2.0)
Assembly statistics	Length	552,713,363	534,638,157	503,815,983
	N50	17,879,143	4,591,336	1,210,516
	L50	9	29	130
	No. of contigs	149	996	4,840
	Largest contig	42,954,611	26,880,362	8,149,722
	GC %	35.62	35.78	35.50
BUSCO results (OrthoDB v10 Insecta database)	Complete (single, duplicated)	97.9% (96.4%, 1.5%)	98.1% (96.9%, 1.2%)	96.7% (95.0%, 1.7%)
	Fragmented	0.6%	0.7%	1.5%
	Missing	1.5%	1.2%	1.8%

We then evaluated the HiFi assemblies of Cflo-J, which had the highest contiguity. *k*-mer-based evaluation showed high base accuracy and completeness of primary contigs [a quality value (QV) = 68.08 and completeness = 97.6%, Supplementary Table 2]. The Merqury spectrum plot showed a single *k*-mer peak in the primary contigs, indicating extremely low heterozygosity (Supplementary Fig. 1a, red line).

*Copidosoma floridanum* males have a haploid genome as with many hymenopteran insect, the single *k*-mer peak of the primary contig was reasonable. We also confirmed the presence of reads included only in the alternate contigs (Supplementary Fig. 1a, blue line). Alternate contigs may be a set of similar sequences that are separated from the primary contig because we utilized the adult male *C. floridanum*, which were identical clones derived from a single egg.

Taxonomic assignments of the contigs were performed to reveal the taxonomic origin of the contigs. The primary and alternate contigs were assigned to Arthropoda, and the remaining contigs were either no-hit or chordata (Supplementary Fig. 1b and c). The mapping rate of no-hit was remarkably low (primary contig, 0.27%; alternate contig, 0.51%), and that of chordata in alternate contigs was also extremely low (0.05%). The assembly of Cflo-US also includes Arthropoda and no-hit (65.3%) contigs as shown in Cflo-J (ENA https://www.ebi.ac.uk/ena/browser/view/GCA_000648655.2, accessed on September 20, 2023). In conclusion, an ideal haploid representation was observed in the primary contigs, and no contig contamination from non-arthropods was observed.

### Gene annotation differences between Cflo-J and Cflo-US

As repetitive sequences may affect gene prediction accuracy (Hoff et al. 2019), these sequences in the HiFi primary contig were investigated using RepeatModeler and RepeatMasker. The repeat sequence was 285.4 Mb, corresponding to 51.65% of the HiFi primary contig, and “Unclassified” accounted for a substantial percentage of the total (34.97%) (Supplementary Table 3). We checked the “Unclassified” ratio described in other studies (Shi et al 2019; Sethuraman et al 2022; Ye et al 2022; Wang et al 2023; Xiao et al 2023) (Supplementary Table 4). As long as searching, few species reached 70% of unclassified repeat sequences in all repeat sequences, although *Dinocampus coccinellae* reached 85%. The high proportion of unclassified repeat sequences suggests that the repeat sequences are unique compared to other wasps.

Before gene annotation, we checked if we could improve BUSCO duplication with purge_dups (Guan et al 2020). We performed purge_dups for primary HiFi contig (purged assembly: Supplementary File 3). We confirmed the reduction of the duplicated ratio (Supplementary Table 5). However, purge_dups has a limited ability for repeat sequences (Guan et al 2020), and may remove repeat sequences which are resolved by Hifiasm. We checked the abundance of repeat sequences before and after performing purge_dups with RepeatModeler/RepeatMasker. Genome size was reduced after purge_dups (about 11Mb: 552713363 to 541712804 bp) and all repeats identified were also reduced (about 9.4 Mb: 285049684 to 275685809 bp). This shows that the reduction in genome size is almost due to the removal of repeat sequences, which may be a true duplicated region resolved by Hifiasm. In this study, the assembly with no purge_dups applied was used.

Gene prediction was performed using several RNA-Seq data and protein dataset combinations. The BRAKER3 using RNA-Seq data and the Arthropoda protein dataset showed the best BUSCO completeness (96.4%, Table 2). The numbers of predicted genes and transcripts are listed in Table 2. BRAKER2 predicted more genes compared to BRAKER3 (Table 2). The gene model with the highest BUSCO completeness (96.4%) (“best gene model”) predicted 10,786 protein-coding genes and 13,886 transcripts. The annotated GFF files are shown in Supplementary File 4. The best gene model was annotated using Fanflow4Insects (Bono et al. 2022). Table 3 shows the results of the searches using Fanflow4Insects for each reference dataset. Of the 10,786 gene models, 10,585 were annotated using GGSEARCH or HMMSCAN. The full annotation results for GGSEARCH and HMMSCAN are presented in Supplementary Table 6.

**Table 2.** BUSCO analysis for the protein set predicted by BRAKER.

	BRAKER3 with RNA seq + Arthropoda protein	BRAKER2 with RNA seq	BRAKER3 with Arthropoda protein	BRAKER2 with Arthropoda protein
Complete (single, duplicated)	96.4% (79.5%, 16.9%)	94% (73.9%, 20.1%)	93.1% (78.8%, 14.3%)	92.5% (82.8%, 9.7%)
Fragmented	0.5%	2.40%	0.70%	1.80%
Missing	3.1%	3.60%	6.20%	5.70%
No. of genes	10,786	18,531	9913	22,781
No. of transcripts	13,886	19,986	12,370	24,487

**Table 3.** Summary of functional annotation results.

Annotation category	Reference	The number of gene count
	Human	8,467
	Mouse	8,291
	*Caenorhabditis elegans*	7,061
Gene annotation by top hit (GGSEARCH)	Fly	8,403
	*Copidosoma floridanum*	9,850
	UniProtKB/Swiss-Prot	9,038
	At least one of the above	10,537
Genes annotated only by HMMSCAN	Pfam domain database	48
All genes annotated at protein level	At least one of the above	10,585
Hypothetical protein	No hit	201
Total		10,786

**Fig. 2.**
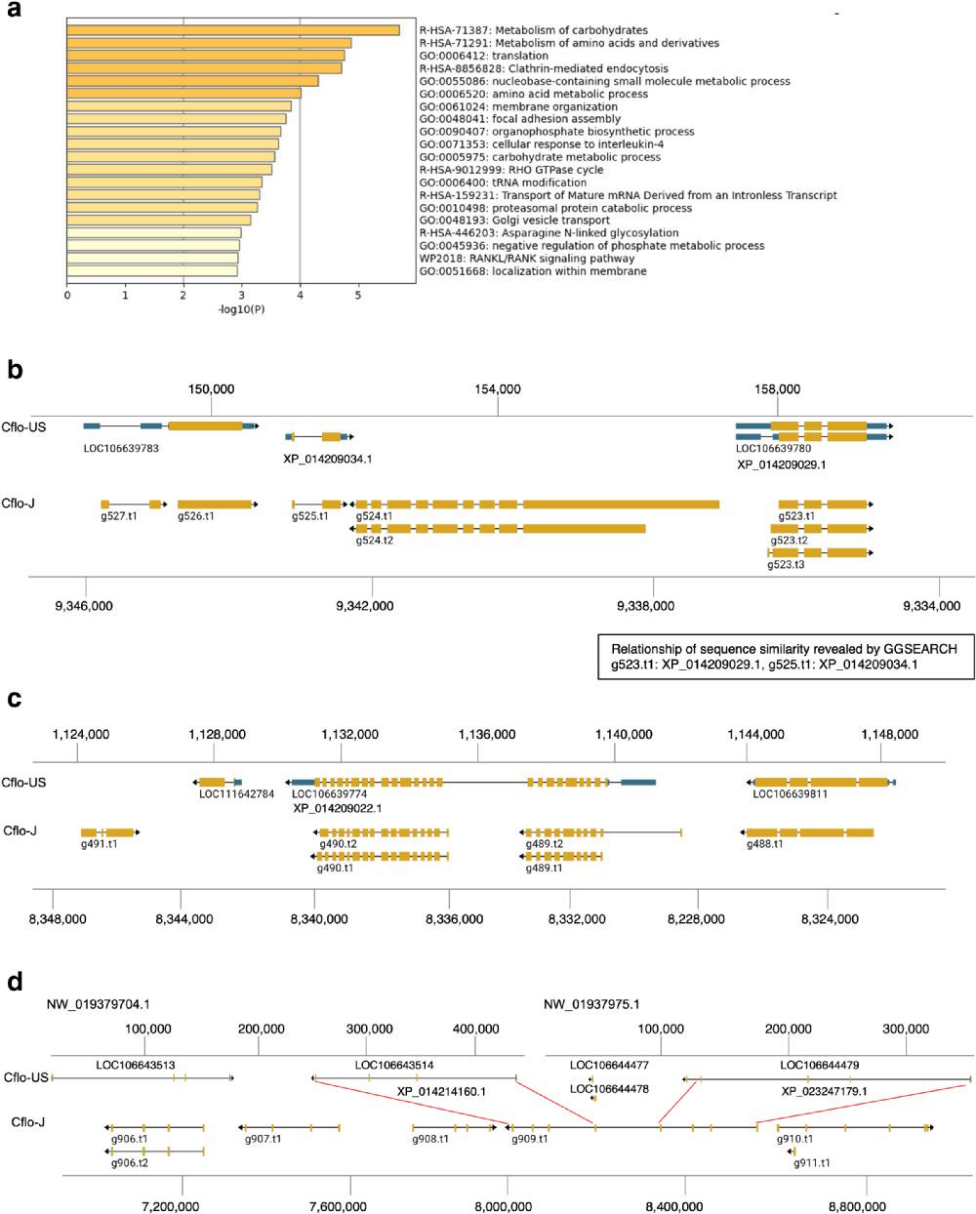
Features of genes with no corresponding proteins to Cflo-US. a) The results of enrichment analysis (Metascape) of 149 transcripts with no corresponding proteins to Cflo-US and with corresponding proteins to human, mouse, *Caenorhabditis elegans*, and fly. Bars are colored differently according to the value of -log10 (*P*-value). b) g524, c) g490, and (d) g909 are shown as representative gene model illustrating the genome annotation differences. Yellow and blue boxes indicate the ORF and UTR, respectively. Red lines indicate the region with similar exon structures.

We evaluated whether annotation differences existed between the Cflo-US assembly and Cflo-J HiFi assembly. We identified 149 transcripts in 115 gene loci with no corresponding proteins to Cflo-US and with corresponding proteins to humans, mouse, *C. elegans*, and fly (Supplementary Table 7). The human gene IDs corresponding to 149 transcripts were used for enrichment analysis (Fig. 2a). “Metabolism of carbohydrates” was the most significantly enriched Gene Ontology (GO) term. In addition, GO terms for amino acid metabolism and translation were enriched.

Excluding gene loci with at least one isoform corresponding to Cflo-US genes, the number of genes as mentioned above was limited from 115 to 100. We investigated why these 100 genes did not hit the Cflo-US gene model. To verify whether corresponding regions of the 100 genes are absent in the Cflo-US, TBLASTN of 100 genes was performed for the genome of Cflo-US. We manually examined hit regions of TBLASTN using NCBI Genome Data viewer. TBLASTN results showed that all 100 genes hit in the Cflo-US genome. Thus, the difference in genome annotation between Cflo-US and Cflo-J is not due to differences in the extent of the sequenced genomic regions. Among 100 genes, 21 genes had no corresponding gene models in Cflo-US assembly, while 79 genes had corresponding gene models in Cflo-US (Supplementary Table 8). To show the characteristics of those gene sets (100 genes, 21 genes, and 79 genes), we performed enrichment analysis by Metascape, and there were significantly enriched GO terms in both gene sets (Supplementary Fig. 2). The gene model g524 was representative example without corresponding gene model of Cflo-US (Fig. 2b). On both sides of g524 are g523 and g525. Amino acid sequences of g523 and g525 have sequence similarity to XP_014209029.1 and XP_014209034.1 in Cflo-US genome, respectively (Supplementary Table 4). Although these genomic position and sequence similarity indicate that the genomic position around g524 is conserved in both genome assemblies, the gene model corresponding to g524 was absent in Cflo-US.

Among 79 genes with corresponding gene model in Cflo-US, TBLASTN results were divided into two groups: genes that hit the single gene model or genes that hit several gene models (two or three) (Supplementary Table 8). In addition, differences in the amino acid length between Cflo-J and Cflo-US were also revealed by the TBLASTN (Supplementary Table 8). g490 was representative example of the gene that hit the single gene model of Cflo-US. TBLASTN showed that the amino acid sequence of g490.t1 had a similarity to XP_014209022.1 of Cflo-US gene model (Supplementary Table 8). The exon structures of XP_014209022.1 were different from g490 of Cflo-J (Fig. 2c). Multiple sequence alignment showed that the amino acid length of XP_014209022.1 of Cflo-US was extremely longer at N-terminal region than the corresponding protein of other species (Supplementary Fig. 3). When BLASTP was adopted, g490.t1 showed high sequence similarity to XP_014209022.1 (*E*-value: 0.0), despite the gene structures were largely different. This indicates that GGSEARCH adopted in this study can detect the genes with extremely different structures of genes. g909 was the representative example as the gene that hit several gene models of Cflo-US (Supplementary Table 8). TBLASTN showed that g909 had the similarity XP_023247179.1 and XP_014214160.1, and these two genes of Cflo-US were distributed in different scaffolds (Fig. 2d). Multiple sequence alignment indicated that XP_023247179.1 and XP_014214160.1 correspond to the first and second half of the amino acid sequence of g909.t1, respectively (Supplementary Fig. 4). The example of g909 suggested that long-read sequencing contributed to the accuracy of genome annotation. TBLASTN also detected the genes that hit more than two gene models on the same scaffold (Supplementary Table 8, e.g. g1061.t1).

In this study, we found genes with extremely different gene annotations between Cflo-US and Cflo-J. This success is attributed to the method used in this study. GGSEARCH, which was adopted in this study, searches for sequence similarity with the subject within 80–120% of the query length. This contributed to the discovery of protein structure differences between Cflo-J and Cflo-US. In addition, highly contiguous genome assembly obtained by long-read sequencing suggested the fragmentation of gene model in Cflo-US genome.

We performed GGSEARCH for proteins in Cflo-US but not in Cflo-J annotation, and found 3,225 proteins (1,803 genes) with corresponding proteins to human, mouse, *C. elegans*, and fly (Supplementary Table 9). This indicates that 3,225 protein sequences have no correspondence with proteins of Cflo-J. We tested whether this implies a lack of gene prediction in the Cflo-J assembly. Using the human gene IDs of Cflo-J and Cflo-US as keys, the annotation results were compared as follows. The 3,225 proteins were clustered as 1,803 human gene IDs, and 1,518 of 1,803 human gene IDs were found in Cflo-J annotation in Supplementary Table 4. This indicates that there is not much lack of gene prediction in the Cflo-J assembly compared with Cflo-US assembly. However, 285 of 1,803 genes were unique in Cflo-US. Enrichment analysis by Metascape for 285 genes showed that the GO term “Assembly of the ORC complex at the origin of replication” was enriched particularly (Supplementary Fig. 5).

### Common features and differences of *vasa* between cflo-j and cflo-US

**Fig. 3.**
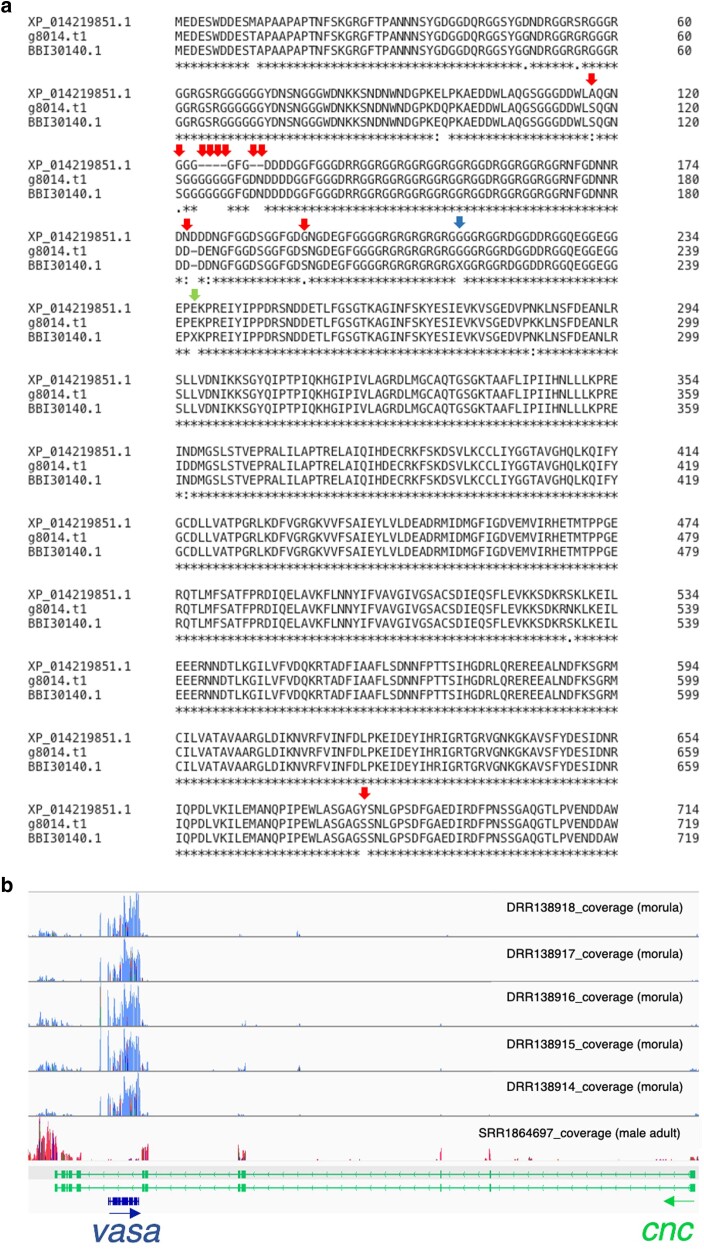
Amino acid sequences genomic position and of *vasa* gene. Comparison of gene annotation between the Cflo-US and Cflo-J HiFi assemblies. a) Comparison of amino acid sequences of the *vasa* gene in the US (XP_014219851.1) and Japanese strains (g8014.t1 and BBI30140.1). g8014.t1 is the transcript ID of *vasa* predicted in Cflo-J assembly, and BBI30140.1 is the protein ID of *vasa* in the Japanese lineage reported by Ohno et al. (2019). Red and green arrows indicate amino acid position that differ between the US and Japanese strains, as Ohno et al. 2019 revealed. Green and blue arrows indicate two amino acid site that were not determined as amino acid sequences in Ohno et al. 2019. b) Genomic position of *vasa* with RNA-Seq read coverages including the information of developmental stages. The CDS regions are shown at the bottom, including the *vasa* and *cap’n’collar* (*cnc*) exon structures. Blue and green arrows indicate the direction of transcription.

Ohno et al. (2019) showed that the amino acid sequence of *vasa* differed between Cflo-J and Cflo-US. The amino acid sequences of Cflo-US (XP_014219851.1) and Cflo-J *vasa* (g8014.t1), including the amino acid sequence (BBI30140.1) determined by Ohno et al. (2019), were compared. We confirmed the differences in amino acid sequences at the N-terminal region between Cflo-US *vasa* (XP_014219851.1) and Cflo-J *vasa* (g8014.t1), as previously reported (Fig. 3a, red and green arrows). There were two amino acids that were not determined as amino acid sequences in Ohno et al. 2019, but the current study could determine that in Cflo-J *vasa* using long-read sequencer (Fig. 3a, green and blue arrows).

We also investigated the genomic position of *vasa* (Fig. 3b). *vasa* were located in an intron between the seventh and eighth exons of *cap’n’collar* (*cnc*), and the directions of transcription were opposite (Fig. 3b, blue and green arrows). This positional relationship was also confirmed in Cflo-US (NCBI Genome Browser: LOC106647823, Location: scaffold6|NW_019379589.1:2,626,762–2,630,760.). Generally, overlapping genes such as *vasa* and *cnc* are negatively selected evolutionarily, whereas overlapping genes are sometimes retained in a species-specific manner (Craig et al. 1997). In addition, overlapping genes are likely to be co-expressed. We confirmed the expression of *vasa* and *cnc* using RNA-seq data in morula and adult males which was used for gene prediction in this study. The expression level of *cnc* was similar in the morula and adult male, however, *vasa* was highly expressed in morula compared to adult males. The soldier larvae of the Cflo-J strain develop faster than those of the Cflo-US strain. Therefore, the expression levels of *vasa* and *cnc* may be related to caste differentiation. Further transcriptome data are needed to prove the cooperation of *vasa* and *cnc* in caste differentiation. Furthermore, the *vasa* genomic positions in other insects were preliminarily examined (Supplementary Table 10), and the positional relationship between *vasa* and *cnc* may be conserved in Hymenoptera. Genomic positional relationship of *vasa* and *cnc* may be related to the oogenesis or embryogenesis in hymenopteran insects. *cnc* is related to the nuclei arrangement of oocyte in *Drosophila melanogaster* (Guichet et al. 2001). This suggests that *cnc* may also be involved in embryogenesis in *C. floridanum*. As described above, *vasa* and *cnc* expression are likely to cooperatively be involved in the caste differentiation of *C. floridanum*, but it is unclear why their positional relationships are conserved across Hymenoptera. The focus on the regulation of expression of these two genes throughout Hymenoptera may provide an opportunity to elucidate the evolutionary process of *vasa*-regulated caste differentiation in *C. floridanum*.

## Conclusion

We obtained a highly contiguous genome assembly of Japanese *C. floridanum*. We showed that long-read sequencing and functional annotation method adopted in this study can highlight the differences in genome annotation between Cflo-J and Cflo-US. Parasitic wasps can control the physiological state of host insects, and their use for pest control is expected (Soldà et al. 2008). Comparison of the genome sequences of Cflo-US and Cflo-J will contribute to the identification of detailed genomic regions relevant to the ecological and physiological characteristics of *C. flolidanum*, leading to a detailed understanding of its parasitic ability against pests.

## Data Availability

All sequencing data (assembled sequences and raw sequence reads) were deposited in the DDBJ under the umbrella BioProject accession number PRJDB16642. Genome assembly from primary contigs was deposited in the DDBJ under the accession numbers BTPR01000001–BTPR01000149. Alternate contigs were deposited in DDBJ under the accession numbers BTPQ01000001–BTPQ01001162. Raw sequence reads were deposited in the DDBJ under accession number DRA017002 (PacBio HiFi reads) and DRA017125 (PacBio Subreads). Gene annotation and functional annotation of protein-coding genes are available at figshare (https://doi.org/10.6084/m9.figshare.24145182.v8). Supplementary Files are available at figshare (https://doi.org/10.6084/m9.figshare.24145182.v8). Scripts of genome assembly, quality check, and gene annotation were also available (https://doi.org/10.6084/m9.figshare.24145182.v8).
